# Brain Age Gap Progression in Individuals at Risk for Neurodegeneration

**Published:** 2026-10-13

**Authors:** Jason Scheffel, Saurabh Sihag

## Abstract

Neuroimaging is a widely used modality for characterizing neurodegeneration in various health conditions. Brain age gap has emerged as a promising biomarker for monitoring brain health from neuroimaging data using machine learning algorithms. Recent studies have leveraged coVariance neural networks (VNN)-driven pipelines based on the advancements in graph signal processing for relatively transparent construction of brain age gap from neuroimaging data. In this paper, we focus on a longitudinal study of VNN-based brain age gap in conjunction with Alzheimer's Progression Score (APS) in individuals that are at a higher risk of neurodegeneration due to family history of Alzheimer's disease. APS is a multimodal marker of disease progression. The longitudinal analysis of APS and VNN-derived brain age gap metrics revealed that a higher risk of disease progression at baseline predicted a larger rate of increase in brain age gap in follow-up visits. Thus, our findings revealed potential stratification in the brain age gap modulated by clinical/biological risks of AD in the at-risk population.

